# *catena*-Poly[[dichloridoiron(II)]-μ-4,4′′-bis­(benzimidazol-1-yl)-1,1′:4′,1′′-terphen­yl]

**DOI:** 10.1107/S1600536810002837

**Published:** 2010-02-06

**Authors:** Hui Li, Zhuangwei Wei, Qiaojuan Gong, Qiuping Han

**Affiliations:** a Department of Applied Chemistry, Yuncheng University, Yuncheng, Shanxi 044000, People’s Republic of China

## Abstract

In the title coordination polymer, [FeCl_2_(C_32_H_22_N_4_)]_*n*_, the Fe^II^ atom lies on a crystallographic twofold axis and a distorted FeCl_2_N_2_ tetra­hedral coordination geometry arises. The complete ligand is generated by crystallographic twofold symmetry, resulting in an infinite one-dimensional architecture along [101].

## Related literature

For background to benzimidazoles as ligands, see: Vijayan *et al.* (2006 ▶).
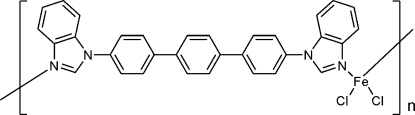


## Experimental

### 

#### Crystal data

[FeCl_2_(C_32_H_22_N_4_)]
*M* *_r_* = 589.29
Monoclinic, 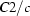

*a* = 14.519 (3) Å
*b* = 14.303 (3) Å
*c* = 12.461 (3) Å
β = 101.94 (3)°
*V* = 2531.6 (9) Å^3^
*Z* = 4
Mo *K*α radiation
μ = 0.84 mm^−1^
*T* = 293 K
0.20 × 0.18 × 0.15 mm

#### Data collection

Rigaku Saturn CCD area-detector diffractometer
Absorption correction: multi-scan (*CrystalClear*; Rigaku/MSC, 2005 ▶) *T*_min_ = 0.846, *T*_max_ = 0.882
9515 measured reflections
2218 independent reflections
1960 reflections with *I* > 2σ(*I*)
*R*_int_ = 0.052

#### Refinement

*R*[*F* ^2^ > 2σ(*F* ^2^)] = 0.049
*wR*(*F* ^2^) = 0.117
*S* = 1.12
2218 reflections
177 parameters
H-atom parameters constrained
Δρ_max_ = 0.35 e Å^−3^
Δρ_min_ = −0.41 e Å^−3^

### 

Data collection: *CrystalClear* (Rigaku/MSC, 2005 ▶); cell refinement: *CrystalClear*; data reduction: *CrystalClear*; program(s) used to solve structure: *SHELXS97* (Sheldrick, 2008 ▶); program(s) used to refine structure: *SHELXL97* (Sheldrick, 2008 ▶); molecular graphics: *SHELXTL* (Sheldrick, 2008 ▶); software used to prepare material for publication: *SHELXTL*.

## Supplementary Material

Crystal structure: contains datablocks I, global. DOI: 10.1107/S1600536810002837/hb5310sup1.cif

Structure factors: contains datablocks I. DOI: 10.1107/S1600536810002837/hb5310Isup2.hkl

Additional supplementary materials:  crystallographic information; 3D view; checkCIF report

## Figures and Tables

**Table 1:** Selected bond lengths (Å)

Fe1—N1	2.076 (2)
Fe1—Cl1	2.2489 (10)

**Fig. 1.**
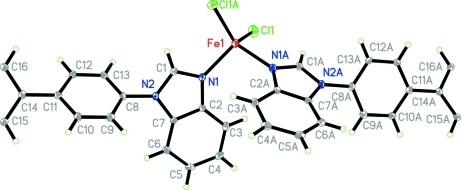
A fragment of a polymeric chain in (I). Displacement ellipsoids are drawn at the 30% probability level and H atoms are shown as small spheres of arbitrary radius.

## supplementary crystallographic information

### Comment

Benzimidazole has been well used in crystal engineering, and a large number of benzimidazole-containing flexible ligands have been extensively studied. However, to our knowledge, the research on benzoimidazole ligands bearing rigid spacers is still less developed.

Single-crystal X-ray diffraction analysis reveals that the title compound crystallizes in the monoclinic space group *C*2/*c*. The geometry of the Fe^II^ ion is surrounded by two benzimidazole rings of distinct L ligands and two chlorine anions, which illustrates a slightly distorted tetrahedral coordination environment (Fig. 1). Notably, as shown in Fig. 2, the four-coordinated Fe^II^ center is bridged by the linear ligand L to form an infinite one-dimensional architecture along crystallographic [101] axis.

### Experimental

A mixture of (CH_3_)_2_CHOH and CHCl_3_ (1:1, 8 ml), as a buffer layer, was carefully layered over a solution of 4,4'-Bis(benzimidazol-1-yl)terphenyl (L, 0.06 mmol) in CHCl_3_ (6 ml). Then a solution of FeCl_2_ (0.02 mmol) in (CH_3_)_2_CHOH (6 ml) was layered over the buffer layer, and the resultant reaction was left to stand at room temperature. After *ca* three weeks, yellow blocks of (I) appeared at the boundary. Yield: ~10% (based on L).

### Refinement

C-bound H atoms were positioned geometrically and refined in the riding-model approximation, with C—H = 0.93Å and *U*_iso_(H) = 1.2*U*_eq_.

The N-bound H atoms were located in a difference map and their positions were freely refined with *U*_iso_(H) = 1.2*U*_eq_(N).

### Crystal data

**Table:**

[FeCl_2_(C_32_H_22_N_4_)]	*F*(000) = 1208
*M**_r_* = 589.29	*D*_x_ = 1.546 Mg m^−^^3^
Monoclinic, *C*2/*c*	Mo *K*α radiation, λ = 0.71073 Å
Hall symbol: -C 2yc	Cell parameters from 2574 reflections
*a* = 14.519 (3) Å	θ = 2.0–27.9°
*b* = 14.303 (3) Å	µ = 0.84 mm^−^^1^
*c* = 12.461 (3) Å	*T* = 293 K
β = 101.94 (3)°	Block, yellow
*V* = 2531.6 (9) Å^3^	0.20 × 0.18 × 0.15 mm
*Z* = 4	

### Data collection

**Table:**

Rigaku Saturn CCD area-detector diffractometer	2218 independent reflections
Radiation source: fine-focus sealed tube	1960 reflections with *I* > 2σ(*I*)
graphite	*R*_int_ = 0.052
Detector resolution: 9 pixels mm^-1^	θ_max_ = 25.0°, θ_min_ = 2.0°
ω scans	*h* = −17→16
Absorption correction: multi-scan (*CrystalClear*; Rigaku/MSC, 2005)	*k* = −16→15
*T*_min_ = 0.846, *T*_max_ = 0.882	*l* = −14→14
9515 measured reflections	

### Refinement

**Table:**

Refinement on *F*^2^	Primary atom site location: structure-invariant direct methods
Least-squares matrix: full	Secondary atom site location: difference Fourier map
*R*[*F*^2^ > 2σ(*F*^2^)] = 0.049	Hydrogen site location: inferred from neighbouring sites
*wR*(*F*^2^) = 0.117	H-atom parameters constrained
*S* = 1.12	*w* = 1/[σ^2^(*F*_o_^2^) + (0.0565*P*)^2^ + 2.1075*P*] where *P* = (*F*_o_^2^ + 2*F*_c_^2^)/3
2218 reflections	(Δ/σ)_max_ = 0.001
177 parameters	Δρ_max_ = 0.35 e Å^−^^3^
0 restraints	Δρ_min_ = −0.41 e Å^−^^3^

### Special details

**Table:**

Geometry. All e.s.d.'s (except the e.s.d. in the dihedral angle between two l.s. planes) are estimated using the full covariance matrix. The cell e.s.d.'s are taken into account individually in the estimation of e.s.d.'s in distances, angles and torsion angles; correlations between e.s.d.'s in cell parameters are only used when they are defined by crystal symmetry. An approximate (isotropic) treatment of cell e.s.d.'s is used for estimating e.s.d.'s involving l.s. planes.
Refinement. Refinement of *F*^2^ against ALL reflections. The weighted *R*-factor *wR* and goodness of fit *S* are based on *F*^2^, conventional *R*-factors *R* are based on *F*, with *F* set to zero for negative *F*^2^. The threshold expression of *F*^2^ > σ(*F*^2^) is used only for calculating *R*-factors(gt) *etc*. and is not relevant to the choice of reflections for refinement. *R*-factors based on *F*^2^ are statistically about twice as large as those based on *F*, and *R*- factors based on ALL data will be even larger.

### Fractional atomic coordinates and isotropic or equivalent isotropic displacement parameters (Å^2^)

**Table:**

	*x*	*y*	*z*	*U*_iso_*/*U*_eq_	
Fe1	0.0000	1.12084 (4)	0.7500	0.0240 (2)	
Cl1	0.02255 (6)	1.20219 (6)	0.90773 (8)	0.0423 (3)	
N2	0.20786 (16)	0.96985 (16)	0.63670 (19)	0.0186 (6)	
N1	0.10013 (17)	1.02547 (17)	0.72169 (19)	0.0207 (6)	
C15	0.4708 (2)	0.8783 (2)	0.2868 (2)	0.0176 (6)	
H15	0.4516	0.8216	0.3115	0.021*	
C11	0.3788 (2)	0.9625 (2)	0.4047 (2)	0.0181 (6)	
C14	0.4398 (2)	0.9609 (2)	0.3250 (2)	0.0185 (6)	
C8	0.2661 (2)	0.9662 (2)	0.5587 (2)	0.0187 (6)	
C13	0.3223 (2)	1.0412 (2)	0.5485 (2)	0.0183 (6)	
H13	0.3231	1.0931	0.5937	0.022*	
C3	0.1067 (2)	0.8921 (2)	0.8545 (2)	0.0213 (7)	
H3	0.0598	0.9141	0.8887	0.026*	
C10	0.3213 (2)	0.8878 (2)	0.4174 (3)	0.0228 (7)	
H10	0.3212	0.8351	0.3736	0.027*	
C1	0.1466 (2)	1.0385 (2)	0.6442 (2)	0.0203 (7)	
H1	0.1381	1.0905	0.5984	0.024*	
C9	0.2649 (2)	0.8892 (2)	0.4924 (3)	0.0233 (7)	
H9	0.2261	0.8387	0.4986	0.028*	
C16	0.4710 (2)	1.0437 (2)	0.2859 (2)	0.0199 (6)	
H16	0.4514	1.1005	0.3098	0.024*	
C7	0.2006 (2)	0.9061 (2)	0.7177 (2)	0.0171 (6)	
C12	0.3767 (2)	1.0395 (2)	0.4721 (2)	0.0184 (6)	
H12	0.4137	1.0913	0.4647	0.022*	
C2	0.1330 (2)	0.9411 (2)	0.7698 (2)	0.0177 (6)	
C5	0.2242 (2)	0.7782 (2)	0.8350 (2)	0.0245 (7)	
H5	0.2559	0.7235	0.8605	0.029*	
C6	0.2490 (2)	0.8247 (2)	0.7494 (2)	0.0212 (7)	
H6	0.2957	0.8027	0.7149	0.025*	
C4	0.1526 (2)	0.8102 (2)	0.8854 (2)	0.0235 (7)	
H4	0.1360	0.7750	0.9412	0.028*	

### Atomic displacement parameters (Å^2^)

**Table:**

	*U*^11^	*U*^22^	*U*^33^	*U*^12^	*U*^13^	*U*^23^
Fe1	0.0229 (4)	0.0202 (4)	0.0323 (4)	0.000	0.0134 (3)	0.000
Cl1	0.0413 (6)	0.0368 (5)	0.0522 (6)	−0.0083 (4)	0.0174 (5)	−0.0199 (4)
N2	0.0184 (13)	0.0194 (13)	0.0205 (13)	0.0010 (10)	0.0099 (10)	0.0014 (10)
N1	0.0213 (14)	0.0203 (14)	0.0226 (13)	0.0014 (11)	0.0097 (11)	0.0035 (11)
C15	0.0176 (16)	0.0168 (15)	0.0187 (14)	−0.0018 (12)	0.0041 (12)	0.0017 (12)
C11	0.0186 (16)	0.0178 (15)	0.0182 (14)	0.0007 (12)	0.0043 (12)	0.0020 (12)
C14	0.0170 (15)	0.0204 (16)	0.0185 (14)	−0.0008 (12)	0.0047 (12)	0.0003 (12)
C8	0.0172 (15)	0.0216 (16)	0.0192 (14)	0.0036 (12)	0.0082 (12)	0.0026 (12)
C13	0.0190 (16)	0.0155 (15)	0.0203 (15)	0.0014 (12)	0.0041 (12)	−0.0030 (12)
C3	0.0202 (16)	0.0266 (17)	0.0182 (15)	−0.0030 (13)	0.0066 (12)	−0.0023 (13)
C10	0.0272 (18)	0.0191 (17)	0.0253 (16)	−0.0050 (13)	0.0125 (14)	−0.0069 (13)
C1	0.0193 (16)	0.0190 (16)	0.0242 (15)	0.0009 (13)	0.0081 (13)	0.0044 (13)
C9	0.0243 (17)	0.0193 (16)	0.0281 (17)	−0.0046 (13)	0.0097 (14)	−0.0021 (13)
C16	0.0224 (16)	0.0167 (15)	0.0218 (15)	−0.0004 (12)	0.0074 (12)	−0.0023 (12)
C7	0.0199 (16)	0.0134 (14)	0.0189 (14)	−0.0024 (12)	0.0057 (12)	0.0013 (12)
C12	0.0183 (16)	0.0171 (15)	0.0200 (14)	−0.0018 (12)	0.0044 (12)	0.0004 (12)
C2	0.0159 (15)	0.0170 (15)	0.0211 (15)	0.0003 (12)	0.0058 (12)	−0.0011 (12)
C5	0.0288 (18)	0.0147 (16)	0.0284 (17)	0.0007 (13)	0.0018 (14)	0.0025 (13)
C6	0.0203 (16)	0.0185 (16)	0.0257 (16)	0.0001 (13)	0.0066 (13)	−0.0037 (13)
C4	0.0315 (18)	0.0207 (16)	0.0181 (15)	−0.0060 (13)	0.0048 (13)	0.0024 (13)

### Geometric parameters (Å, °)

**Table:**

Fe1—N1^i^	2.076 (2)	C13—H13	0.9300
Fe1—N1	2.076 (2)	C3—C4	1.362 (4)
Fe1—Cl1^i^	2.2489 (10)	C3—C2	1.384 (4)
Fe1—Cl1	2.2489 (10)	C3—H3	0.9300
N2—C1	1.342 (4)	C10—C9	1.364 (4)
N2—C7	1.381 (4)	C10—H10	0.9300
N2—C8	1.415 (4)	C1—H1	0.9300
N1—C1	1.300 (4)	C9—H9	0.9300
N1—C2	1.388 (4)	C16—C16^ii^	1.349 (6)
C15—C15^ii^	1.373 (6)	C16—H16	0.9300
C15—C14	1.383 (4)	C7—C6	1.375 (4)
C15—H15	0.9300	C7—C2	1.379 (4)
C11—C10	1.386 (4)	C12—H12	0.9300
C11—C12	1.389 (4)	C5—C6	1.367 (4)
C11—C14	1.461 (4)	C5—C4	1.398 (4)
C14—C16	1.392 (4)	C5—H5	0.9300
C8—C13	1.368 (4)	C6—H6	0.9300
C8—C9	1.375 (4)	C4—H4	0.9300
C13—C12	1.358 (4)		
			
N1^i^—Fe1—N1	97.86 (13)	C9—C10—C11	121.9 (3)
N1^i^—Fe1—Cl1^i^	120.53 (7)	C9—C10—H10	119.1
N1—Fe1—Cl1^i^	99.90 (7)	C11—C10—H10	119.1
N1^i^—Fe1—Cl1	99.90 (7)	N1—C1—N2	113.6 (3)
N1—Fe1—Cl1	120.53 (7)	N1—C1—H1	123.2
Cl1^i^—Fe1—Cl1	117.69 (6)	N2—C1—H1	123.2
C1—N2—C7	106.3 (2)	C10—C9—C8	119.3 (3)
C1—N2—C8	125.0 (2)	C10—C9—H9	120.4
C7—N2—C8	128.6 (2)	C8—C9—H9	120.4
C1—N1—C2	105.1 (2)	C16^ii^—C16—C14	121.72 (17)
C1—N1—Fe1	121.4 (2)	C16^ii^—C16—H16	119.1
C2—N1—Fe1	133.50 (19)	C14—C16—H16	119.1
C15^ii^—C15—C14	121.33 (17)	C6—C7—C2	122.9 (3)
C15^ii^—C15—H15	119.3	C6—C7—N2	131.2 (3)
C14—C15—H15	119.3	C2—C7—N2	105.9 (2)
C10—C11—C12	117.0 (3)	C13—C12—C11	121.8 (3)
C10—C11—C14	122.0 (3)	C13—C12—H12	119.1
C12—C11—C14	121.0 (3)	C11—C12—H12	119.1
C15—C14—C16	117.0 (3)	C7—C2—C3	120.7 (3)
C15—C14—C11	122.2 (3)	C7—C2—N1	109.1 (2)
C16—C14—C11	120.8 (3)	C3—C2—N1	130.2 (3)
C13—C8—C9	120.3 (3)	C6—C5—C4	122.1 (3)
C13—C8—N2	119.2 (3)	C6—C5—H5	118.9
C9—C8—N2	120.5 (3)	C4—C5—H5	118.9
C12—C13—C8	119.7 (3)	C5—C6—C7	115.8 (3)
C12—C13—H13	120.1	C5—C6—H6	122.1
C8—C13—H13	120.1	C7—C6—H6	122.1
C4—C3—C2	117.1 (3)	C3—C4—C5	121.3 (3)
C4—C3—H3	121.5	C3—C4—H4	119.3
C2—C3—H3	121.5	C5—C4—H4	119.3
			
N1^i^—Fe1—N1—C1	139.0 (3)	N2—C8—C9—C10	179.6 (3)
Cl1^i^—Fe1—N1—C1	16.0 (2)	C15—C14—C16—C16^ii^	−0.1 (5)
Cl1—Fe1—N1—C1	−114.6 (2)	C11—C14—C16—C16^ii^	178.6 (3)
N1^i^—Fe1—N1—C2	−40.2 (2)	C1—N2—C7—C6	−177.4 (3)
Cl1^i^—Fe1—N1—C2	−163.2 (3)	C8—N2—C7—C6	3.3 (5)
Cl1—Fe1—N1—C2	66.2 (3)	C1—N2—C7—C2	0.4 (3)
C15^ii^—C15—C14—C16	−0.3 (5)	C8—N2—C7—C2	−178.9 (3)
C15^ii^—C15—C14—C11	−179.0 (3)	C8—C13—C12—C11	−1.3 (4)
C10—C11—C14—C15	−23.9 (4)	C10—C11—C12—C13	1.1 (4)
C12—C11—C14—C15	155.5 (3)	C14—C11—C12—C13	−178.3 (3)
C10—C11—C14—C16	157.5 (3)	C6—C7—C2—C3	−3.6 (5)
C12—C11—C14—C16	−23.2 (4)	N2—C7—C2—C3	178.3 (3)
C1—N2—C8—C13	53.0 (4)	C6—C7—C2—N1	177.6 (3)
C7—N2—C8—C13	−127.8 (3)	N2—C7—C2—N1	−0.5 (3)
C1—N2—C8—C9	−125.7 (3)	C4—C3—C2—C7	2.0 (4)
C7—N2—C8—C9	53.5 (4)	C4—C3—C2—N1	−179.4 (3)
C9—C8—C13—C12	0.3 (4)	C1—N1—C2—C7	0.4 (3)
N2—C8—C13—C12	−178.4 (3)	Fe1—N1—C2—C7	179.6 (2)
C12—C11—C10—C9	0.1 (5)	C1—N1—C2—C3	−178.3 (3)
C14—C11—C10—C9	179.5 (3)	Fe1—N1—C2—C3	1.0 (5)
C2—N1—C1—N2	−0.1 (3)	C4—C5—C6—C7	1.4 (4)
Fe1—N1—C1—N2	−179.48 (19)	C2—C7—C6—C5	1.8 (4)
C7—N2—C1—N1	−0.2 (3)	N2—C7—C6—C5	179.3 (3)
C8—N2—C1—N1	179.1 (3)	C2—C3—C4—C5	1.1 (4)
C11—C10—C9—C8	−1.2 (5)	C6—C5—C4—C3	−2.9 (5)
C13—C8—C9—C10	0.9 (5)		

Symmetry codes: (i) −*x*, *y*, −*z*+3/2; (ii) −*x*+1, *y*, −*z*+1/2.
